# Norepinephrine prevents hypotension in older patients under spinal anesthesia with intravenous propofol sedation: a randomized controlled trial

**DOI:** 10.1038/s41598-023-48178-2

**Published:** 2023-11-29

**Authors:** Hyungtae Kim, Sooho Lee, Won Uk Koh, Jooyeon Cho, Sung Wook Park, Keon Sik Kim, Young-Jin Ro, Ha-Jung Kim

**Affiliations:** 1 Department of Anesthesiology and Pain Medicine, Asan Medical Center, University of Ulsan College of Medicine, 88, Olympic-ro 43-gil, Songpa-gu, Seoul, 05505 Korea; 2 Department of Anesthesiology and Pain Medicine, Catholic Kwandong University, College of Medicine, International St. Mary’s Hospital, Incheon, Korea; 3 Department of Anesthesiology and Pain Medicine, Kyung Hee University Hospital, Kyung Hee University College of Medicine, Seoul, Korea

**Keywords:** Medical research, Outcomes research, Clinical trial design

## Abstract

Reducing hypotension is crucial as hypotension is the most common side effect of spinal anesthesia, and in older patients with various comorbidities, it can lead to fatality. We hypothesized that continuous infusion of norepinephrine could effectively prevent hypotension in older patients undergoing hip surgery under spinal anesthesia with propofol sedation. The study randomly assigned patients aged ≥ 70 years to either a control (Group C, n = 35) or a norepinephrine group (Group N, n = 35). After spinal anesthesia, continuous infusion of propofol and normal saline or norepinephrine was initiated. The number of hypotensive episodes, the primary outcome, as well as other intraoperative hemodynamic events and postoperative complications were compared. In total, 67 patients were included in the final analysis. The number of hypotensive episodes was significantly higher in Group C than in Group N (*p* < 0.001). Furthermore, Group C required a greater amount of fluid to maintain normovolemia (*p* = 0.008) and showed less urine output (*p* = 0.019). However, there was no difference in postoperative complications between the two groups. Continuous intravenous infusion of prophylactic norepinephrine prevented hypotensive episodes, reduced the requirement of fluid, and increased the urine output in older patients undergoing unilateral hip surgery under spinal anesthesia with propofol sedation.

Clinical trial registration number: KCT0005046 (https://cris.nih.go.kr). IRB number: 2020-0533 (Institutional Review Board of Asan Medical Center, approval date: 13/APR/2020).

## Introduction

Hypotension is the most common side effect of spinal anesthesia (SA), especially in older individuals. High-segment sensory nerve block and advanced age are major risk factors for hypotension after SA^1^. Propofol, which is widely used for sedation, acts as a vasodilator by decreasing sympathetic activity, and can reduce blood pressure when injected intravenously. Therefore, if propofol is injected intravenously for sedation during surgery in patients receiving SA, the incidence of hypotension may increase. In addition, in older patients with various comorbidities, postoperative complications resulting from intraoperative hypotension (IOH) can lead to fatalities. In the past, phenylephrine was commonly used for the management of SA-induced hypotension^2–5^. Recently, however, norepinephrine has been described as an effective alternative to phenylephrine for the prevention and treatment of hypotension during SA for cesarean delivery^6–11^. Relatively few studies have evaluated the effectiveness of continuous infusion of norepinephrine in preventing IOH in older patients^12,13^. Therefore, in this study, we aimed to investigate whether norepinephrine administration can effectively prevent hypotension in older patients aged ≥ 70 years undergoing unilateral primary hip surgery under SA with propofol sedation. Moreover, we evaluated the effect of continuous norepinephrine infusion on the intraoperative hemodynamic events and postoperative outcomes.

## Results

**Figure 1:**
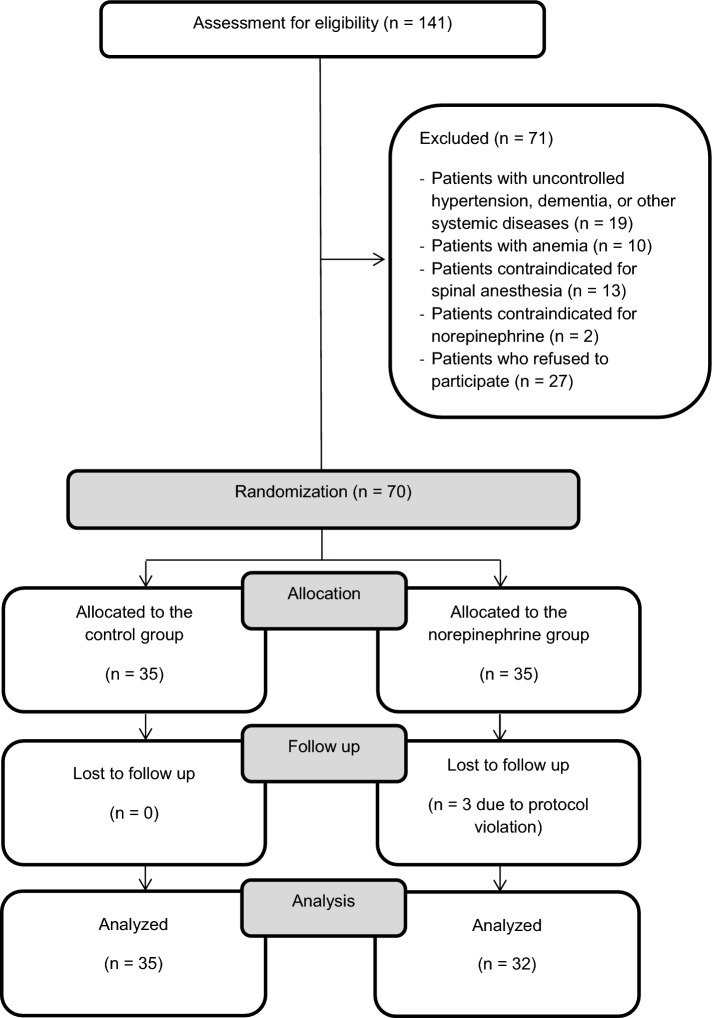
Flow diagram of this study.

In total, 67 patients were included in the final analysis (Fig. 1). All data were expressed as means ± standard deviations or medians [interquartile ranges]. There was no difference in the demographic data, co-existing disease, or preoperative laboratory data between the two groups, except for the albumin level (Table 1).

**Table 1:** Demographic data, co-existing diseases, and preoperative laboratory data of the study participants.

	Group C (n = 35)	Group N (n = 32)	*p*-value
Demographic data			
Age (years)	78.7 ± 6.2	79.3 ± 6.1	0.678
Male/female (n)	12/23 (34%/66%)	8/24 (25%/75%)	0.407
Height (cm)	158.0 ± 9.1	156.0 ± 8.0	0.336
Weight (kg)	54.8 ± 12.2	55.8 ± 12.9	0.751
Body mass index (kg/m^2^)	22.1 ± 4.3	22.9 ± 4.5	0.430
ASA 1/2/3 (n)	0/25/10 (0%/71%/29%)	0/24/8 (0%/75%/25%)	0.742
Co-existing diseases			
Diabetes mellitus (n)	10 (29%)	8 (25%)	0.742
Hypertension (n)	21 (60%)	15 (47%)	0.282
Ischemic heart disease (n)	6 (17%)	4 (13%)	0.736
Cerebrovascular accident (n)	1 (3%)	3 (9%)	0.342
Pulmonary disease (n)	4 (11%)	9 (28%)	0.123
Renal disease (n)	4 (11%)	3 (9%)	1.000
Others (n)	31 (89%)	26 (81%)	0.501
Preoperative laboratory data			
Hemoglobin (g/dL)	12.2 ± 1.7	12.2 ± 1.4	0.949
White blood cell (10^3^/µL)	7.3 [5.8–8.9]	6.8 [5.7–9.0]	0.720*
Platelet (10^9^/µL)	216.1 ± 71.4	214.7 ± 65.2	0.935
K^+^ (mmol/L)	4.3 ± 0.5	4.2 ± 0.4	0.379
Ca^2+^ (mmol/L)	9.2 [9.0–9.6]	9.1 [8.8–9.6]	0.174*
Aspartate aminotransferase (IU/L)	23.6 ± 8.0	26.4 ± 11.0	0.235
Alanine aminotransferase (IU/L)	18.7 ± 13.7	20.8 ± 16.4	0.577
Creatinine (mg/dL)	0.83 [0.64–1.04]	0.75 [0.66–0.90]	0.763*
Glomerular filtration rate	80.0 [61.0–87.0]	79.5 [61.5–87.0]	0.826*
Albumin (g/dL)	3.7 [3.3–4.0]	3.4 [3.2–3.6]	0.017*
C-reactive protein (mg/dL)	0.22 [0.10–3.14]	0.26 [0.10–1.08]	0.844*

Data are presented as means ± standard deviations, numbers (%), or medians [interquartile ranges]. ASA, American Society of Anesthesiology; Group C, control group; Group N, norepinephrine group.

*Wilcoxon's rank sum test.

Table 2 presents the surgery-related data of the two groups. We found no significant differences in the type of surgery, anesthesia time, operation time, propofol infusion time, and intraoperative blood loss. However, the two groups showed significant differences in the total amount of crystalloid solution and urine output during surgery. The control group (Group C) required a greater amount of crystalloid solution to maintain normovolemia based on the value of the pleth variability index (PVi) compared to the norepinephrine group (Group N) (Group C, 795.7 ± 312.3 mL; Group N, 579.7 ± 336.9 mL; *p* = 0.008). Urine output was also greater in Group N than in Group C (Group N, 70.0 [42.5–155.0] mL; Group C, 40.0 [0.0–80.0] mL; *p* = 0.019). No adverse events were observed related to the peripheral vein route of norepinephrine administration.

**Table 2:** Surgery and anesthesia-related data.

	Group C (n = 35)	Group N (n = 32)	*p*-value
Diagnosis (n)			0.401
Fracture	15 (43%)	17 (53%)	
Non-fracture	20 (57%)	15 (47%)	
Type of surgery (n)			0.120
Bipolar hemiarthroplasty	12 (34%)	17 (53%)	
Total hip arthroplasty	23 (66%)	15 (47%)	
Anesthesia level (n)			0.484*
T8	16 (46%)	18 (56%)	
T10	18 (51%)	12 (38%)	
T11	1 (3%)	2 (6%)	
Anesthetic time (min)	137.1 ± 23.9	139.2 ± 27.1	0.734
Operation time (min)	72.1 ± 20.6	75.1 ± 25.4	0.583
Propofol infusion time (min)	99.9 ± 23.8	103.3 ± 24.3	0.596
Intraoperative blood loss (mL)	200 [100–300]	300 [100–400]	0.629^†^
Intravenous fluid volume (mL)	795.7 ± 312.3	579.7 ± 336.9	0.008
Urine output (mL)	40.0 [0.0–80.0]	70.0 [42.5–155.0]	0.019^†^

Data are presented as means ± standard deviations, numbers (%), or medians [interquartile ranges]. Group C, control group; Group N, norepinephrine group.

*Fisher’s exact test.

^†^Wilcoxon's Rank Sum Test.

The primary outcome and the number of hypotensive episodes during surgery were significantly higher in Group C than in Group N (Group C, 7 [3–15]; Group N, 0 [0–0.5]; *p* < 0.001, Table 3). The number of patients who experienced one or more hypotensive episodes during surgery was also greater in Group C (Group C, 31, 89%; Group N, 8, 25%). Although the times to the first injection of phenylephrine showed no difference, the rescue phenylephrine doses were greater in Group C. However, the two groups demonstrated no difference in the occurrence of hypertension, tachycardia, and bradycardia. Among the nine patients who experienced hypertension in Group N, there was no need to reduce the dose of norepinephrine. Additionally, there were no differences in hypotensive events during the postoperative 1-h period between the two groups.

**Table 3:** Intraoperative and immediate postoperative outcome data, including adverse episodes, events, and physician interventions.

	Group C (n = 35)	Group N (n = 32)	*p*-value
Intraoperative adverse events			
Hypotension	31 (89%)	8 (25%)	< 0.001
Number of hypotensive episodes	7 [3–15]	0 [0–0.5]	< 0.001*
Hypertension	5 (14%)	9 (28%)	0.165
Number of hypertensive episodes	0 [0–0]	0 [0–1]	0.260^†^
Bradycardia	7 (20%)	3 (9%)	0.310
Number of bradycardia episodes	0 [0–0]	0 [0–0]	0.232^†^
Tachycardia	2 (6%)	1 (3%)	1.000
Number of tachycardia episodes	0 [0–0]	0 [0–0]	0.612^†^
Interventions			
Rescue phenylephrine dose (µg)	700 [300–1500]	0 [0–50]	< 0.001^†^
Time to first injection of phenylephrine (min)	24 [15–33]	37 [10–102]	0.330^†^
Postoperative hypotension (1 h postoperatively)			
Hypotension	10 (29%)	7 (22%)	0.529
Number of hypotensive episodes	0 [0–1]	0 [0–0]	0.532^†^

Data are presented as number (%) or median [interquartile ranges]. Group C, control group; Group N, norepinephrine group.

*Poisson test.

^†^Wilcoxon’s rank sum test.

**Figure 2:**
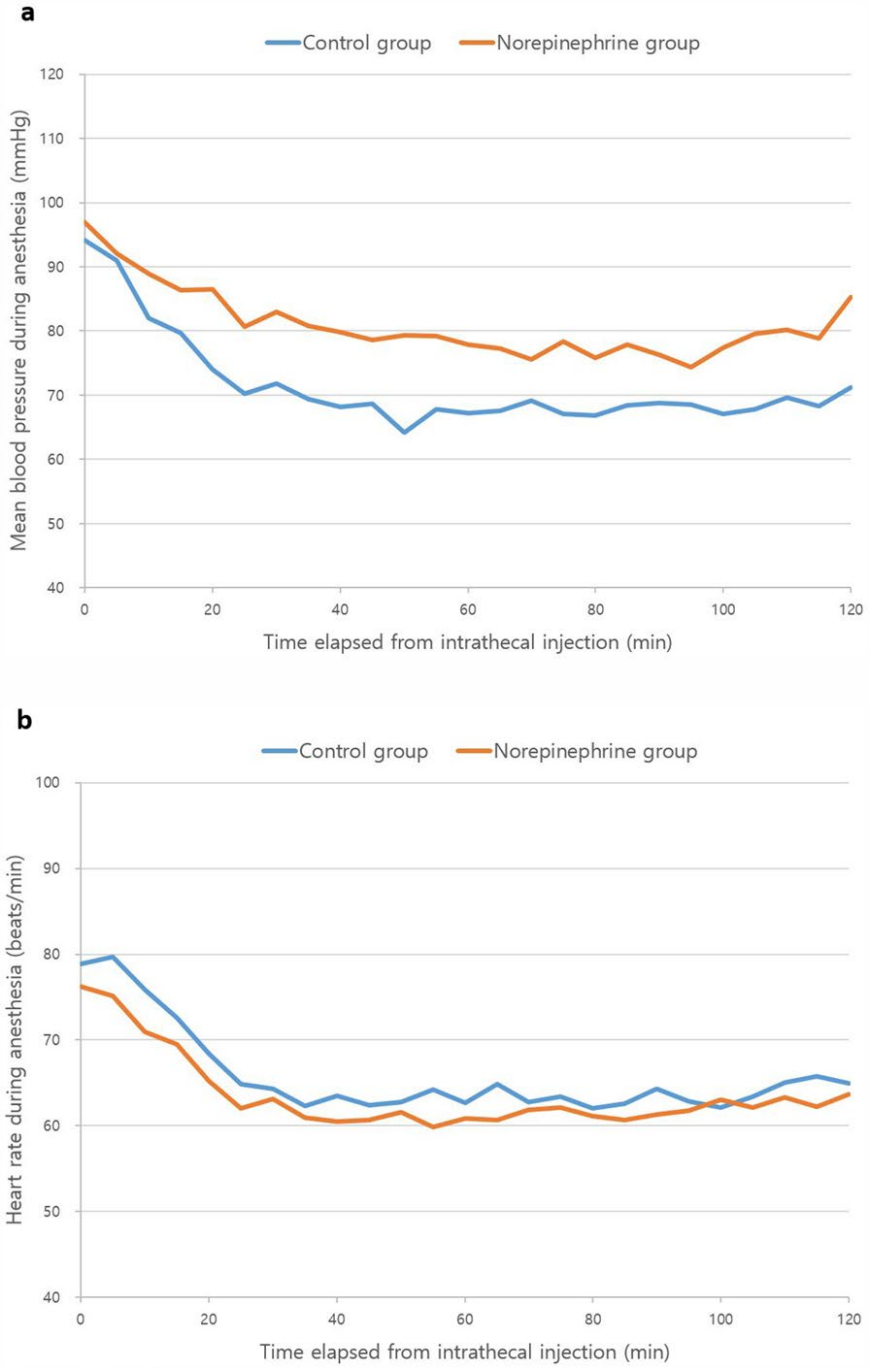
Changes in the mean blood pressure and heart rate during the intraoperative period (**a**): Mean blood pressure during anesthesia (mmHg). (**b**): Heart rate during anesthesia (beats/min).

Linear mixed model showed that the change of the mean blood pressure (MBP) was affected by both the group effect and time effect (all *p*-values < 0.001, Fig. 2a), which demonstrated that the MBP decreased over time, and the change of the MBP was significantly greater in Group C compared to that in Group N. The interaction of group and time was not significant (*p* = 0.054). In the analysis of the change in the heart rate (HR) using a linear mixed model, time was the only significant factor identified (*p* < 0.001, Fig. 2b). In terms of postoperative morbidity and the length of hospital stay, there were no significant differences between the two groups (Table 4 and Supplementary Table 1).

**Table 4:** Postoperative complications and length of stay.

	Group C (n = 35)	Group N (n = 32)	*p*-value
Cardiovascular complication	2 (5.7%)	1 (3.1%)	1.000
Neurologic complication	0 (0.0%)	0 (0.0%)	1.000
Respiratory complication	3 (8.6%)	2 (6.3%)	1.000
Delirium	4 (11.4%)	1 (3.1%)	0.358
Renal dysfunction	4 (11.4%)	2 (6.3%)	0.675
Others	6 (17.7%)	3 (9.4%)	0.480
Length of stay (day)	4 [4–10]	4 [4–11]	0.830

Data are presented as numbers (%) and medians [interquartile ranges].

Group C, control group; Group N, norepinephrine group.

## Discussion

We found that infusing norepinephrine under SA with propofol sedation during hip surgery in patients aged ≥ 70 years was effective in decreasing the number of hypotensive episodes per patient and the incidence of hypotension during SA with intravenous propofol infusion.

Lower extremity surgery represents 24% of all interventions among patients aged ≥ 75 years^14^. Particularly, hip fracture surgery in older patients is reportedly associated with high rates of morbidity and mortality^15^. Although SA is often preferred to general anesthesia owing to several benefits for patients with hip fracture^15,16^, hypotension is a relatively frequent complication during SA. SA-induced hypotension is more common in older patients, with a reported incidence of 65–75%^17,18^. The frequent incidence of hypotension following SA in the older patients is a notable anesthetic concern, as this patient population often has multiple comorbidities and is vulnerable to organ hypoperfusion due to reduced functional reserve^19^. Hypotension results from the sympathetic blockade after induction of SA, which sequentially decreases systemic vascular resistance and cardiac output (CO)^20^.

As the degree of hypotension is proportional to the height of the sympathetic nerve block^20^, it is important to limit the block height to the greatest extent possible. Therefore, in our study, a small amount of bupivacaine (10 mg) with 15 µg of fentanyl was injected intrathecally for SA, and the block height of SA was adjusted to approximately T10 to reduce the risk for hypotension. Nevertheless, hypotensive events occurred in 89% of patients in Group C. Patients undergoing surgery under SA often ask to be sedated during surgery to relieve anxiety, even though there is no surgery-related pain. Agents, such as propofol and dexmedetomidine, are commonly used for sedation during SA. Notably, propofol has been demonstrated to be significantly associated with IOH, likely owing to its vasodilating effect, especially when compared to dexmedetomidine^21–23^. In contrast, Shin et al. highlighted a higher incidence of hypotension in the postanesthetic care unit among patients administered dexmedetomidine, compared to those receiving propofol^23^. Additionally, the onset and offset times of the sedative effect of dexmedetomidine were longer than those of propofol^21^. Considering these characteristics, propofol offers certain advantages over dexmedetomidine. However, when administering propofol sedation during SA, it is advisable to minimize the dose of propofol and maintain light sedation^24^. Although low-dose propofol was injected to maintain light sedation with bispectral index (BIS) between 60 and 80 in our study, the use of propofol may still have contributed to the high incidence of hypotension^25^.

Several strategies, including intravenous fluid administration and vasopressors, have been considered to prevent the occurrence of hypotension after SA induction. Prophylactic administration of crystalloid before SA is effective at preventing hypotension, at least in some patients^26,27^. However, it should be considered that the decrease in systemic vascular resistance cannot be prevented by the administration of fluids alone, and that the increase in preload and CO may be insignificant despite the administration of fluids in older individuals. In addition, large amounts of fluid administration can be dangerous in older patients with poor cardiac or pulmonary reserves^28^. Therefore, it is necessary to restore systemic vascular resistance using vasopressors, such as alpha agonists^6^. Hence, in our study, intravenous co-hydration with only 300 mL of crystalloid and continuous infusion of norepinephrine were initiated immediately after the completion of intrathecal injection^29^.

The use of alpha-agonists to treat hypotension associated with SA has been extensively studied in the field of obstetrics. Phenylephrine, a pure alpha1-adrenergic receptor agonist that has no direct effect on HR, showed a beneficial effect during SA for cesarean delivery^2–5^. However, phenylephrine is associated with a dose-dependent decrease in the HR and CO^30,31^. This has stimulated the recent investigation of norepinephrine as an alternative option^6–11^. Norepinephrine acts as an alpha-receptor agonist and exhibits weak beta-receptor agonist activity. Consequently, norepinephrine has a lower tendency to decrease HR, resulting in improved maintenance of CO when compared with phenylephrine in patients undergoing cesarean section^11^. Nevertheless, caution should be exercised when extrapolating these findings from obstetric patients to older patients, as the former are typically younger and have relatively fewer comorbidities. In our study, which included older patients, Group N showed a significantly lower incidence of hypotension, and there was a trend toward a lower incidence of bradycardia compared to Group C. A previous study also noted a higher tendency of bradycardia without statistical significance when phenylephrine was infused after SA in older patients^19^. Based on these findings, we cautiously predict that, similar to obstetric patients, norepinephrine will effectively prevent hypotension during SA with propofol sedation, while reducing the occurrence of bradycardia compared to phenylephrine in older patients.

Although more than 100 definitions of hypotension are mentioned in the literature, Salmasi et al. found that there was no advantage to using relative over absolute thresholds^32^. In addition, pain and presurgical anxiety may cause an overestimation of BP readings in hip fracture patients. Hence, this study defined hypotensive episodes based on absolute thresholds rather than patient-specific baselines. Moreover, a previous study identified that the increased time with MBP < 65 mmHg or any exposure to MBP < 55 mmHg was significantly associated with moderately or highly elevated postoperative risk^33^. In our study, which used an absolute threshold as the criterion for hypotension, the exposure time to hypotension in Group C would have been longer, which may also have increased postoperative complication risks. However, despite the difference in the incidence of hypotension between the two groups, there was no disparity in postoperative clinical outcomes. The latter could be explained by the immediate treatment of hypotension in our study protocol. Based on these results, we conclude that both prevention and prompt treatment of hypotension are important; however, considering its frequency in older patients, prevention appears to be the more rational approach. Therefore, continuous infusion of norepinephrine should be considered in older patients receiving SA with propofol sedation.

This study had several limitations. First, PVi values were measured during spontaneous breathing. PVi is a measure of the variations in the pulse oximeter waveform over respiratory cycles in non-invasive and continuous manner. When mechanical ventilation is employed, PVi can be considered reliable^34^. However, PVi clearly showed a significant association with the hemodynamic changes in spontaneous breathing volunteers^35^. Second, we used a fixed dose of 0.05 µg/kg/min of norepinephrine. Hasanin et al. previously suggested that the optimal dose of norepinephrine infusion for preventing SA-induced hypotension was 0.05 µg/kg/min, and that there was no advantage in using the highest dose of 0.075 µg/kg/min^36^. However, as the responses of the patients to norepinephrine may vary, clinicians can adjust the dose of norepinephrine based on the individual responses. If the infusion dose of norepinephrine is adjusted based on individual responses, the hemodynamic variables could be more stable than those in this study. Furthermore, there might be concerns regarding tissue ischemia when administering norepinephrine via a peripheral vein in our study protocol. However, a previous study demonstrated that there was no significant morbidity associated with norepinephrine infusion via the peripheral line^37^. Therefore, our protocol could be safely applied in real clinical settings with minimal concern. Finally, the distribution of patients with and without hip fracture between the two groups was not identical. Given the distinct characteristics of patients with and without hip fracture, the incidence of IOH might have varied between the two groups. However, as statistical analysis revealed no significant difference in the proportion of patients with hip fracture to those without hip fracture, this disparity likely had a minimal impact on the study results.

In conclusion, the present study showed that prophylactic norepinephrine continuous intravenous infusion prevented hypotensive episodes, reduced the occurrence of hypotension and the requirement of fluid, and increased the urine output in older patients undergoing unilateral hip surgery under SA with propofol sedation.

## Methods

This single-center, prospective, randomized controlled trial study was performed in a tertiary center in Seoul, Republic of Korea. After receiving approval from the Institutional Review Board of Asan Medical Center (approval date: April 13, 2020), this trial was registered on the Clinical Research Information Service (http://cris.nih.go.kr, KCT0005046, 21/05/2020). Written informed consent was obtained from all patients for participation, and this study was conducted in accordance with the Declaration of Helsinki.

### Study population and preparation

All older patients scheduled for unilateral primary hip surgery in the lateral position between 2020 August and 2021 June in the host institution were considered eligible for the study. Among them, we included patients who met the following criteria: (1) American Society of Anesthesiologists physical status class 1–3, and (2) age ≥ 70 years. We excluded patients with uncontrolled hypertension, hyperthyroidism, dementia, or symptomatic coronary disease; hemoglobin levels < 10 g/dL; a previous history of allergy to propofol, fentanyl, or bupivacaine; and those who were contraindicated for SA, including coagulopathy, severe aortic stenosis, severe mitral stenosis, and active infection on the lumbar region. Patients who refused to participate in this study or for whom norepinephrine was contraindicated were also excluded.

All included patients were randomly allocated into Group C (n = 35) and Group N (n = 35). Randomization was conducted using a computer-generated randomization program (https://randomization.com) by the corresponding author. Based on the randomization table, nurses who were not involved in this study were informed about the study drug just before the surgery and prepared it as follows: Group C, normal saline 0.3 mL/kg/h; Group N, norepinephrine (norepinephrine 2 mg [Norpin, Hwa-Seong, Korea] mixed in dextrose 5% in water 198 mL) 0.3 mL/kg/h (0.05 µg/kg/min). Neither the anesthesiologists performing the anesthesia nor the patients were aware of the allocated group.

### Preoperative management

The preoperative management was not strictly controlled in this study. However, according to our center's preoperative management protocol for surgical patients, all patients fasted from midnight on the day of surgery. Meanwhile, a maintenance volume of fluid was administered. In patients with hip fracture, pain management was provided during the preoperative period. The patients were instructed to remain at bed rest for stabilization, and if their pain score using numerical rating scale was > 4 points, tramadol hydrochloride 50 mg was administered as a first-line treatment, followed by hydromorphone hydrochloride 1 mg as a secondary treatment. Premedication was not administered to any of the patients preoperatively.

### Anesthesia and surgery

After patients arrived in the operating room, standard monitoring, including non-invasive blood pressure, electrocardiography, and pulse oximetry was initiated. Non-invasive blood pressure (NIBP) was measured in the contralateral arm to the surgical site with 2.5-min intervals throughout the surgery, while electrocardiography, and pulse oximetry were monitored continuously. To maintain normovolemia, PVi (Radical-7®, Masimo Corp., Irvine, CA, USA), a dynamic index of fluid responsiveness, was also continuously monitored on ipsilateral arm to the surgical site. Oxygen was supplied at 5–6 L/min via a simple facemask.

An intravenous peripheral line was accessed at the ipsilateral hand or forearm, and the patients were positioned into their lateral decubitus position. The standard practice was to position patients in a lateral decubitus position with the surgical site downward. However, if the patients expressed discomfort regarding the position, they were placed in a lateral decubitus position with the surgical site upward. SA was performed with hyperbaric bupivacaine 10 mg and fentanyl 15 µg, using 25-gauge needle after skin disinfection. Then, the patients were returned to the supine position, and the insertion of the foley catheter was performed. Intravenous co-hydration with 300 mL of crystalloid was initiated immediately after completion of the intrathecal injection. The success and level of SA was examined on both the left and right sides at 5 min after the intrathecal injection. As all surgeries were conducted in the lateral decubitus position, patients were positioned laterally with the surgical site facing upward. Norepinephrine or normal saline and propofol were connected, and the continuous infusion of norepinephrine or normal saline was initiated through the inserted peripheral line. Propofol was also infused using a target-controlled infusion system (effect-site concentration 1.0–1.5 µg/mL), and was adjusted to maintain a BIS between 60 and 80 and modified observer’s alertness/sedation scale of 3 (responds only after name is called loudly and/or repeatedly). The intraoperative volume status was monitored using PVi values. Considering that the patients were spontaneously breathing and referencing the results of a previous study, fluid was administered to maintain PVi < 19%^35^. Infusion of propofol and norepinephrine was immediately stopped once all surgical procedures were finished.

### Definition of hemodynamic events and management

Hypotension was defined as MBP < 65 mmHg. When a hypotensive event occurred, phenylephrine 100 µg was injected intravenously as a rescue drug, regardless of the group. Measurements of NIBP were performed until 1 h postoperatively, during which time phenylephrine 100 µg was also administered in case of hypotension. Hypertension was defined as systolic blood pressure (SBP) > 160 mmHg, or an increase in MBP > 20% from the baseline value. Baseline MBP (MBP_BL_) was defined as the average of three NIBP values measured in the general ward or emergency room the day before surgery when the patient was in a stable condition. If a hypertensive event occurred, continuous infusion of the study drug was discontinued, and nicardipine 5 µg/kg was injected when the SBP was > 160 mmHg. After the return of MBP within the normal range (80% of MBP_BL_ < MBP < 120% of the MBP_BL_), continuous infusion of the study drug was restarted. When hypertensive events occurred twice, the infusion rate of the study drug was reduced by half. In cases of tachycardia (HR > 120 beats/min) and bradycardia (HR < 50 beats/min), esmolol 0.5 mg/kg and atropine 0.5 mg were administered, respectively.

### Outcome measures and data collections

The primary outcome of this study was the number of hypotensive episodes that occurred during surgery. The secondary outcomes were other hemodynamic events during surgery and postoperative complications during the hospitalization. Postoperative complications included cardiovascular complications (i.e., acute coronary syndrome, congestive heart failure, hypotension, and arrhythmia), neurologic complications (i.e., transient ischemic accident and cerebrovascular accident), respiratory complications (i.e., pneumonia, pulmonary edema, pleural effusion, and desaturation), delirium, renal dysfunction (i.e., acute kidney injury based on Kidney Disease: Improving Global Outcomes criteria), and other complications.

### Statistical analysis

No prior study has clearly identified hypotensive episodes in older patients undergoing SAwith propofol sedation. Therefore, we determined the sample size using Poisson means. The Poisson means were derived from the retrospective review of our clinical experiences. There were 4.75 hypotensive episodes during a single surgery in older patients who underwent hip surgery under SA with propofol sedation without any continuous infusion of vasopressor, whereas there were 1.67 hypotensive episodes with the continuous infusion of norepinephrine. However, as we had only limited experience on the preventive use of norepinephrine, to ensure a sufficient number of patients, we assumed a Poisson mean of double that of 1.67, which is 3.34, for the treatment group. The calculated sample size, with α = 0.05 and power = 80% using a two-sample and two-sided equality test, was 32 patients in each group. Therefore, after considering possible dropouts, we decided to assign 35 patients into each group.

The Poisson test was used to analyze the number of hypotensive episodes. Other continuous variables were analyzed using Student’s *t*-test or Wilcoxon rank-sum test as appropriate, and categorical variables were compared using Fisher’s exact test. In addition, the linear mixed model was applied to evaluate the longitudinal changes of MBP and HR. In the model, we tested group and time effects and interactions of group and time. All analyses were performed using SAS®, version 9.4 (SAS Institute Inc., Cary, NC, USA) was used. A *p*-value < 0.05 was considered statistically significant.

### Supplementary Information

Supplementary Table S1.

## Data Availability

The datasets used and analyzed in the current study are available from the corresponding author on reasonable request.
