# Supplementary material for: Norepinephrine prevents hypotension in older patients under spinal anesthesia with intravenous propofol sedation: a randomized controlled trial
Source: Sci Rep. 2023 Nov 29;13:21009. doi: 10.1038/s41598-023-48178-2 (PMC10686984; doi:10.1038/s41598-023-48178-2)
Supplement: Supplementary file 1 — Supplementary Table S1. [file 41598_2023_48178_MOESM1_ESM.docx]

Supplementary Table 1. Detailed postoperative complications

| Cardiovascular complications | |
| --- | --- |
| Group C | Hypotension (n=2) |
| Group N | Stress induced cardiomyopathy (n=1) |
| Respiratory complications | |
| Group C | Desaturation (n=1), Pneumonia (n=2) |
| Group N | Pneumonia (n=2) |
| Other complications | |
| Group C | Constipation (n=1), diarrhea (n=2), elevated liver enzymes (n=2), fever of unknown origin (n=1) |
| Group N | Difficult urination (n=1), elevated liver enzymes (n=1), hip dislocation (n=1) |

Group C, control group; Group N, norepinephrine group.
